# The Intermediate Filament Network in Cultured Human Keratinocytes Is Remarkably Extensible and Resilient

**DOI:** 10.1371/journal.pone.0002327

**Published:** 2008-06-04

**Authors:** Douglas Fudge, David Russell, Dan Beriault, Whitney Moore, E. Birgitte Lane, A. Wayne Vogl

**Affiliations:** 1 Department of Cellular & Physiological Sciences, University of British Columbia, Vancouver, Canada; 2 Department of Integrative Biology, University of Guelph, Guelph, Canada; 3 Cancer Research United Kingdom (UK) Cell Structure Research Group, College of Life Sciences, University of Dundee, Dundee, Scotland; Dalhousie University, Canada

## Abstract

The prevailing model of the mechanical function of intermediate filaments in cells assumes that these 10 nm diameter filaments make up networks that behave as entropic gels, with individual intermediate filaments never experiencing direct loading in tension. However, recent work has shown that single intermediate filaments and bundles are remarkably extensible and elastic in vitro, and therefore well-suited to bearing tensional loads. Here we tested the hypothesis that the intermediate filament network in keratinocytes is extensible and elastic as predicted by the available in vitro data. To do this, we monitored the morphology of fluorescently-tagged intermediate filament networks in cultured human keratinocytes as they were subjected to uniaxial cell strains as high as 133%. We found that keratinocytes not only survived these high strains, but their intermediate filament networks sustained only minor damage at cell strains as high as 100%. Electron microscopy of stretched cells suggests that intermediate filaments are straightened at high cell strains, and therefore likely to be loaded in tension. Furthermore, the buckling behavior of intermediate filament bundles in cells after stretching is consistent with the emerging view that intermediate filaments are far less stiff than the two other major cytoskeletal components F-actin and microtubules. These insights into the mechanical behavior of keratinocytes and the cytokeratin network provide important baseline information for current attempts to understand the biophysical basis of genetic diseases caused by mutations in intermediate filament genes.

## Introduction

Intermediate filaments are a diverse family of cytoskeletal proteins that assemble into 10 nm diameter filaments in cells[1]. These filaments form a dense network throughout the cytoplasm of most animal cells, and in mammals, they are also found within the tough, epidermally-derived material alpha-keratin, which makes up structures such as hairs, horns, and claws[2]. Knockout studies[3]–[6] and several characterized human genetic diseases[7] demonstrate that cells lacking their usual complement of intermediate filaments can be mechanically fragile, suggesting that intermediate filaments are important for maintaining the mechanical integrity of cells and tissues.

In spite of their importance to the mechanical integrity of cells, the mechanical properties of individual intermediate filaments and how they function within cytoskeletal networks in vivo are not well understood. Intermediate filaments in cells have been assumed to be stiff and fairly inextensible like their counterparts in hard keratins [8]–[10], but recent in vitro studies on single intermediate filaments and bundles suggest that they may be quite soft and remarkably extensible, stretching up to strains of 250%, or 3.5 times their original length before breaking[11]–[13]. Other in vitro studies have examined the mechanical properties of semi-dilute gels formed from suspensions of intermediate filaments[14]–[17]. These experiments demonstrate that intermediate filament gels are softer, more extensible, and exhibit more extreme strain hardening than gels made from F-actin or microtubules.

While the tensile mechanics of single intermediate filaments and the mechanics of intermediate filament gels are not inconsistent with one another, emphasizing one or the other paints a very different picture of the mechanical function of intermediate filaments in cells and the design of the metazoan cytoskeleton in general. For example, a focus on the tensile properties of single filaments leads to questions about the morphology of the cytoskeleton and the mechanical conditions that might lead to intermediate filaments being loaded in tension and the kinds of deformations they typically experience. In contrast, a focus on the properties of semi-dilute gels assumes that intermediate filaments contribute to cell elasticity via entropic mechanisms in which individual filaments and filament bundles are never loaded directly in tension. Which approach is more relevant to the in vivo condition likely depends on the magnitude of cell deformation. At small cell strains, intermediate filament are likely to be found in a tortuous conformation, and therefore entropic gel models are appropriate. At larger strains, however, individual filaments and bundles in the network could be pulled taut, in which case the tensile properties would be more relevant.

In this study, we aimed to answer the following questions: 1. What happens to the morphology of the intermediate filament network as cells are deformed? 2. Is it possible to deform cells to the point where intermediate filaments are straightened and presumably loaded in tension? 3. If so, are these deformations physiologically relevant, i.e. do the cells survive? 4. At what magnitude of cell strain, if any, does the intermediate filament network show signs of damage?

To answer these questions, we needed to be able to impose large magnitude strains on cells and simultaneously monitor the morphology of the intermediate filament network. We accomplished the former requirement by constructing a custom uniaxial stretching device that can be mounted on an upright epifluorescence microscope and can deform living adherent cells to strains as high as 160%. To visualize the network, we worked with a line of human keratinocytes that expresses a GFP-tagged version of keratin 14 (K14) that incorporates into the cytokeratin intermediate filament network. The dense network of K5/K14 filaments in these cells and their ability to adhere to and grow on collagen-coated silastic membranes make these cells an excellent model for this kind of study[18]. We were also able to fix and embed cells on a similar cell stretching device, which allowed us to visualize the intermediate filament network in both stretched and relaxed cells using TEM.

## Results and Discussion

**Figure 1:**
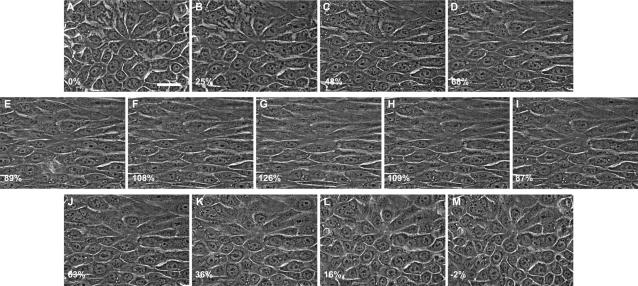
Phase contrast images of NEB-1 keratinocytes undergoing incremental uniaxial strain. Average uniaxial strain is reported in the lower left corner of each panel. Some loss of adhesion occurs at such high strains, which is why cell strain is slightly negative when the rubber substrate is returned to its relaxed state (M). Scale bar = 50 µm.

**Figure 2:**
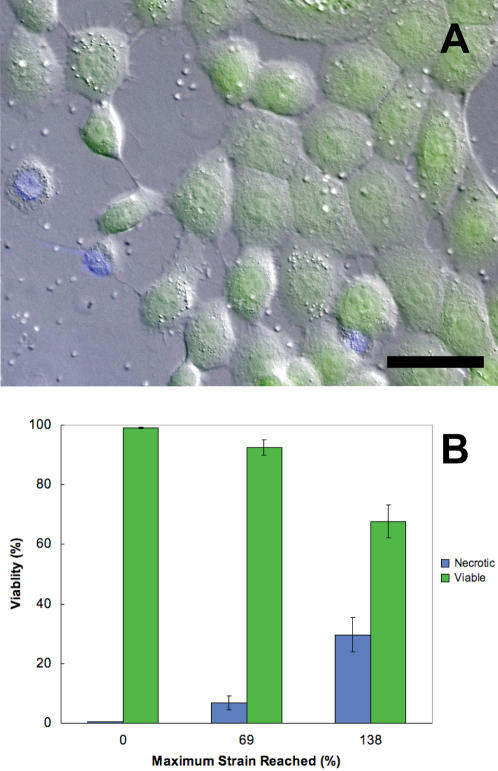
Viability tests using the vital inclusion dye fluorescein diacetate (green) and the vital exclusion dye DAPI (blue) demonstrate that most keratinocytes survived even the most extreme uniaxial stretch treatments used in this study. The cells shown in (A) were extended to an average maximum cell strain of 133% and then returned to the relaxed state for viability staining. Scale bar = 50 µm. (B) Average cell viability and necrosis (±SE) measured as a function of maximum cell strain reached.

Keratinocytes remained attached to the membrane during and after the stretching protocol and showed only minor loss of adhesion after large-scale uniaxial deformations (Fig. 1). One way of measuring the loss of adhesion between the cells and the silastic substrate is to measure the amount of cell compression after the membrane is returned to its relaxed state. The average compression strain for cells after a maximum strain of 126% was −2%, which suggests that loss of adhesion was minimal. In addition to remaining attached at such high strains, most keratinocytes also retained their structural integrity and remained viable, as measured using the vital inclusion dye fluorescein diacetate and the vital exclusion dye DAPI (Fig. 2). Viability decreased significantly (p<0.001) with increasing cell strain, but remarkably, most cells still survived even the highest strains we subjected them to.

**Figure 3:**
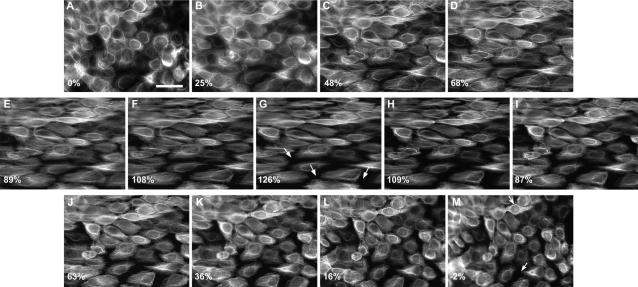
Fluorescent images of NEB-1 K14-GFP keratinocytes undergoing incremental uniaxial strain. Average uniaxial strain is reported in the lower left corner of each panel. These images demonstrate that the cytokeratin network is remarkably robust and can withstand dramatic uniaxial strains without rupturing. In some cases, keratin networks in adjacent cells separated (arrows in panel G), but this corresponded to loss of intercellular adhesion rather than a mechanical failure of the keratin network. The only visible sign of potential damage to the keratin network manifested as keratin bundles that took on a tortuous or wavy appearance after the cells were relaxed back to their resting length (arrowheads). Scale bar = 50 µm.

**Figure 4:**
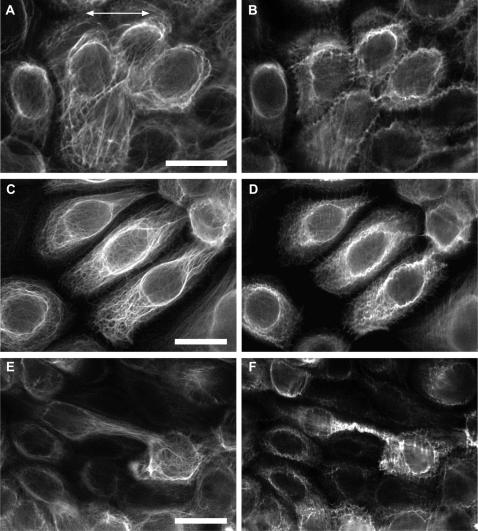
High magnification fluorescent images of NEB-1 K14-GFP cells before (A, C, and E) and after (B, D, and F) the cells were strained to an average maximal strain of 126%. The main effects of this extreme uniaxial strain were increased bundling of the keratin filaments, and an increase in bundle tortuosity. Direction of stretch is indicated by the arrow in panel (A). Scale bars are 20 µm (A) and 25 µm (C) and 25 µm (E).

Following individual cells as they were stretched on an epifluorescent microscope revealed that the intermediate filament network is extensible and resilient like the keratinocytes themselves. In stretching experiments in which cells were deformed uniaxially in increments of approximately 20% cell strain up to a maximum of 126% and then back to the resting state again, the intermediate filament network deformed passively along with the cell (Fig. 3). No evidence of network or bundle rupture was observed during these trials. Even after being subjected to the maximum strain of 126%, the network appeared only slightly distorted after the cells were returned to the resting state. Network disruption after the cells were relaxed was visible as an increase in the “waviness” of intermediate filament bundles (Fig. 4). Another effect that occurred after large-scale strain was an increase in intermediate filament bundling, an effect that was also observed by Russell et al.[19] when they subjected keratinocytes to a cyclic strain regime.

The increase in waviness was likely due to compression of bundles during relaxation of the membrane back to its original, un-deformed state. Compression may have arisen via four mechanisms, none of which are mutually exclusive. First, some compression almost certainly occurred via a partial loss of cell adhesion during stretching, but as described above, this would only contribute a compressional strain of about 2% on average. In addition, we consistently observed the appearance of wavy bundles at points during the incremental relaxation phase when cells were obviously still experiencing significant overall tension and not compression. Second, it is also possible that intermediate filament bundles pulled free from their desmosomal anchors at the cell periphery, but we saw no evidence of this. There were some instances where cells detached from their neighbors and the network recoiled as a result (Fig. 3g), but even in these cases, the intermediate filaments appeared to maintain their connections with the cell periphery. A third possibility is that the intermediate filaments within bundles were plastically deformed to a new resting length by the stretch protocol. This explanation is consistent with two studies that have demonstrated that intermediate filaments are capable of plastic deformation in vitro[11], [12]. We attempted to quantify the length of intermediate filament bundles before, during, and after waviness-inducing stretch regimes, but a lack of fiducial markers on the bundles precluded us from doing this accurately. A fourth possibility is that filaments within bundles slid past one another during stretching, but did not slide back during cell relaxation. While this explanation is plausible if bundles are made up of discontinuous filaments, it is less likely if single continuous filaments within bundles span large distances across the cell and anchor firmly in desmosomes and/or hemidesomomes.

**Figure 5:**
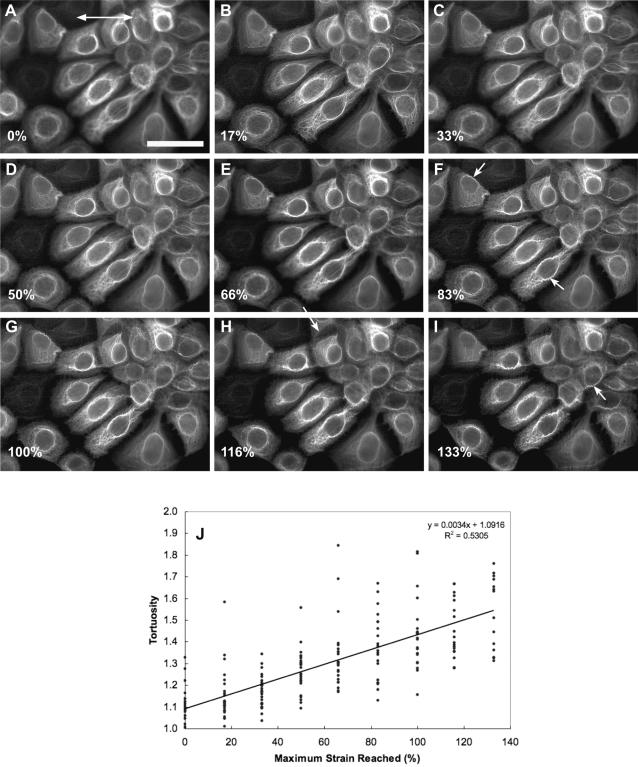
To measure the strain level that leads to keratin bundles adopting a wavy appearance, we loaded NEB-1 K14- GFP keratinocytes to increasingly greater maximal strains and took fluorescent images of the keratin network after the rubber substrate was returned to the unstrained state. Average uniaxial cell strain reached is reported in the lower left corner each panel. Signs of network damage (arrowheads) appeared after cell strains as low as 50%, but became more widespread and obvious after cells were loaded to strains greater than 83%. Direction of stretch is indicated by the arrow in panel (A). Scale bar = 25 µm. (J) The tortuosity of bundles oriented parallel to the stretch axis increased significantly with the magntidue of uniaxial stretch to which the cells were subjected.

All cells that remained firmly attached to the silastic membrane during the incremental stretch trials exhibited an increase in intermediate filament bundle waviness after returning to the resting state (Figs. 3 and 4). To measure the degree of cell strain that induced waviness, we carried out stretch-relax trials in which cells were imaged in the relaxed state after being subjected to an escalating strain regime (Fig. 5). We found that some bundles became wavy after strains of only 50%, with increasing degrees of strain transforming more and more bundles. Most of the variability in the strain at which different bundles became wavy can likely be explained by two variables-the initial degree of slack in the bundle in the relaxed state, and the orientation of the bundle relative to the stretch direction. For example, a bundle that was initially almost taut and oriented parallel to the stretch direction would take on a wavy morphology after stretching to a smaller strain than a bundle that had more slack initially and was oriented at an oblique angle. We were unable to measure bundle lengths directly, but we were able to quantify bundle tortuosity (defined as contour length of a bundle segment divided by the end to end length)[20] in relaxed cells as a function of maximum strain reached, and found that bundles became significantly more tortuous (regression p value<0.001) after the cells were stretched to greater strains (Fig. 5j). This result is consistent with the possibility that intermediate filaments undergo plastic deformation and/or slippage within bundles during large scale stretching events.

**Figure 6:**
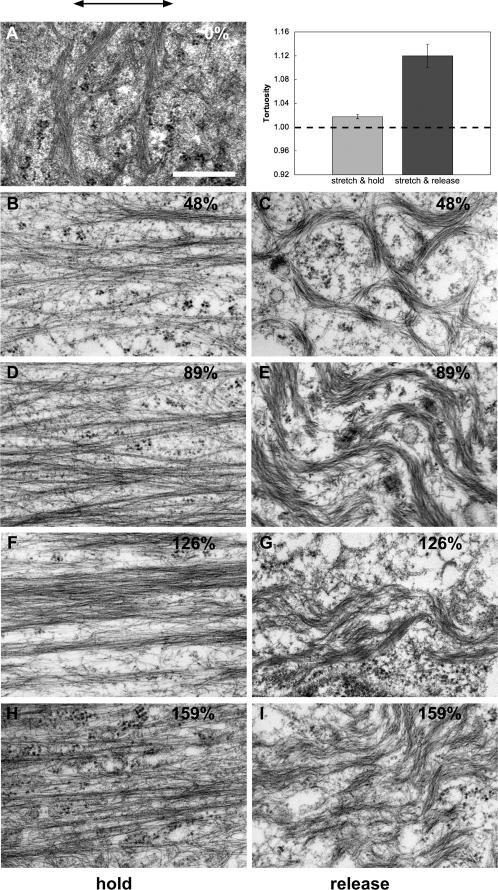
TEM images of keratin intermediate filaments from keratinocytes that experienced a variety of mechanical treatments before being fixed and embedded. A) Control cells that were grown on silastic membranes and embedded without receiving any stretching treatment. All other images are from cells that were either stretched and embedded in the stretched state, or stretched and then unstretched and embedded in the relaxed state. Average maximum strain value is indicated in the upper right of each panel. The direction of stretch is indicated by the arrow above panel (A). Scale bar = 400 nm. (inset) A comparison of the tortuosity of bundles from stretched cells and cells that were stretched and relaxed demonstrates that bundles are straightened by the uniaxial strains we subjected them to.

We used transmission electron microscopy (TEM) to examine in more detail the morphology of individual intermediate filaments and bundles as a function of cell stretch (Fig. 6). Other researchers have used TEM to examine cytoskeletal morphology after mechanical loading of cells[19], but the data presented in Fig. 6 are unique in that they are the first TEM images of cells that were fixed and embedded in the stretched state. Control cells that were never stretched exhibited slightly curved bundles that were randomly oriented (Fig. 6a), as one would expect from previous work[18]. Cells embedded in the stretched state exhibited individual intermediate filaments and bundles that appeared dramatically straightened (Fig. 6b, d, f, and h) parallel to the direction of uniaxial cell strain. In cells that were fixed and embedded after being stretched and then released, bundles appeared wavy as seen in the fluorescence trials, further suggesting that the bundles experienced compression forces during relaxation of the cells. The tortuosity of bundles from cells that were fixed and embedded in the stretched state was significantly lower (p = 0.018) than the tortuosity of bundles from cells that had been stretched and relaxed (Fig. 6j). Furthermore, the average value for bundles oriented parallel to the stretch axis in stretched cells is close to 1.0, which is the tortuosity of a perfectly straight filament. These images and data strongly suggest that intermediate filaments are loaded directly in tension at the strains tested. Kreplak et al.[21] found a dramatic decrease in intermediate filament diameter in vitro when they were loaded to failure and imaged using AFM, but due to extensive filament bundling, we were unable to quantify any potential changes in intermediate filament diameter. Although the maximum uniaxial strains we achieved were far higher than have been reported before, they are still far below the strain at which individual intermediate filaments are likely to break or even decrease dramatically in diameter. Furthermore, the fact that many intermediate filaments have a certain degree of “slack” in unstrained cells means that intermediate filament strain in almost all cases lagged significantly behind cell strain.

Most work on intermediate filament mechanics in cells has focused on in vitro filament networks that behave as soft entropic gels in which single filaments and bundles are never loaded directly in tension[22], [23]. Our TEM results demonstrate that IFs are taut and therefore likely to be loaded in tension at large uniaxial strains. Because the tensile mechanics of single intermediate filaments are characterized by stiffness values that are several orders of magnitude higher[24] than the stiffness of entropic gels (MPa vs. kPa), the direct loading of filaments in tension likely results in a dramatic increase in cell stiffness, even when the concentration of water in the gel is accounted for. Our results do not contradict previous work on intermediate filament gels, but they do suggest that entropic gel models may be inadequate to explain the behavior and mechanical function of intermediate filaments at large cell strains.

The appearance of wavy bundles in cells that were stretched and then released presented an opportunity to infer something about the mechanical properties of intermediate filament bundles and the surrounding cytoplasm. When filaments embedded in an elastic medium are compressed from their ends, they tend to buckle with a characteristic wavelength that is dependent on the bending rigidity of the filaments and the elastic modulus of the surrounding medium[25], [26], according to the following equation:
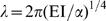
where λ is the buckling wavelength, EI is the bending rigidity of the filaments, E is the elastic modulus of the filaments, I is the 2^nd^ moment of area of the filaments, and α is the elastic modulus of the surrounding medium. From an analysis of TEM images of buckled bundles, we found that the average buckling wavelength was 1.7±0.2 µm (SE) and the average number of filaments in each bundle section was 6.7±1.3 (SE). From the latter measurement, we estimated the total load-bearing cross sectional area of a typical bundle, assuming that bundles were cylindrical in shape, and made up of 10 nm diameter intermediate filaments that were hexagonally packed (i.e. with a packing efficiency of 0.91). We then used this adjusted area (3207 nm^2^) to calculate an effective radius for the bundle (31.9 nm) that we could use to calculate the second moment of area of the bundle using the equation I = πr^4^/4. This equation is for a solid cylinder and so will underestimate the second moment of area somewhat, but not as much as it would be over-estimated by ignoring the spaces between adjacent filaments. Assuming an elastic modulus for intermediate filament bundles of 7 MPa[11], we calculate the stiffness of the surrounding cytoplasm to be about 1 kPa, which is a value that is in good agreement with previous work on cell mechanics[27], [28]. Alternatively, If we assume that intermediate filaments are as stiff as previously assumed in textbooks (2 GPa)[8], [9], the predicted stiffness of the surrounding cytoplasm is 300 kPa, which is not in good agreement with the literature. These measurements support the emerging view of intermediate filaments as soft and extensible reinforcing elements in cells.

We are not the first to describe buckling waves in intermediate filament bundles. Yoon et al.[29] described propagating waves in cytokeratin bundles within live epithelial cells. These waves should not be confused with the intermediate filament “squiggles” described in another paper by the same author[30]. Although they do not provide an analysis of the wavelength of these propagating waveforms, from their figures they appear to be roughly 2.8 µm, which is quite close to the average value of 2.5 µm we measured from our fluorescence images. These values are higher than the wavelength values we obtained from TEM micrographs (1.7 µm) most likely because the limited resolution of the light microscope skewed the data toward waveforms that were larger and therefore more easily detected and measured. The close agreement between Yoon et al.'s data and ours suggests that the buckling behavior of cytokeratin bundles is the same regardless of the source of the compression force on the bundle. For example, in the Yoon study buckling was inhibited by metabolic poisons, which implies that active movements of structures in the cytoplasm occasionally compress keratin bundles to the point of buckling. In our study, the buckling came about via compression of bundles that had taken on a longer resting length after stretching and therefore were compressed when the rest of the cell returned to its previous dimensions.

We would like to emphasize that the results presented here are from a single immortalized cell line, and that future work on other cell lines as well as primary cultures will be needed before our findings can be taken as representative for keratinocytes. In summary, our results suggest that the intermediate filament network in keratinocytes behaves in a manner that is consistent with the dramatic extensibility and elasticity of intermediate filaments in vitro and can survive uniaxial cell strains as high as 100% with little obvious damage. Viability data suggest that keratinocytes are able to survive the kinds of dramatic cell strains that lead to IF bundle straightening and buckling. Electron microscopy of stretched cells suggests that intermediate filaments may be loaded directly in tension at large strains, whereas at low strains, they are more likely to behave as an entropic gel. Lastly, analysis of intermediate filament bundle buckling after stretch-relax trials supports the emerging view of intermediate filaments as relatively soft filaments that are two to three orders of magnitude less stiff than the other two major cytoskeletal filaments, F-actin and microtubules. The dramatic extensibility of living keratinocytes is sensible when one considers that the overlying cornified epidermal layers that they give rise to are also compliant and highly extensible, at least when fully hydrated[31]. These kinds of insights into the mechanical capabilities and limits of wild type keratinocytes will be vital in future efforts to understand the biophysical basis of genetic diseases that are caused by mutations in intermediate filaments, such as the skin-blistering disease epidermolysis bullosa simplex.

## Materials and Methods

### Cell culture

NEB-1 cells expressing wild-type keratins[32] were immortalised using HPV16. These cells were transfected with a GFP-K14 construct that was expressed from a pEGFP-N1 construct. Transfection was carried out using electroporation and cells were selected using G418 (neomycin). Single cell cloning was carried out to generate a clonal population of cells. All experiments described here were carried out on cells between passage 10 and 20 post-immortalisation. Cell lines were cultured in 75% DMEM/25% Ham's F12 medium, containing 10% fetal calf serum (FCS) and additional growth supplements hydrocortisone (0.4 mg/ml), transferrin (5 mg/ml), lyothyronine (2×10^−11^ M), adenine (1.9×10^−4^ M), insulin (5 mg/ml), and EGF (10 mg/ml). These cell lines are fibroblast feeder cell independent and were cultured at 37°C in 5% CO_2_.

### Mechanical stretch

Cells were seeded onto 34×14 mm rectangles of collagen IV coated silicone rubber membranes (Flexcell International, USA) and grown to 80% confluence. Uniaxial stretching of cells was carried out using a custom-built device in which a rectangular piece of silicone rubber could be clamped in place and stretched using a digital micrometer with a non-rotating spindle (Mitutoyo, Japan). To visualize cells adhered to the silicone membrane, the stretching device was mounted on an upright Leica DM-LA epifluorescent microscope (Leica Microsystems, Germany). After a strip with adherent cells was clamped in the device, cells were covered with growth media plus 20 mM HEPES buffer (to keep pH stable outside the incubator) and covered with a 9×18 mm coverslip. Fresh media was added to one side of the coverslip and drawn off from the other side with a piece of filter paper as required during stretching of the membrane.

Two kinds of acute cell straining protocols were employed on the epifluorescent microscope-incremental stretches, and stretch-relax trials. In incremental stretches, the traveling end of the rubber strip was extended in increments of 25% of the resting length using the micrometer. Cells were stretched at a rate of 0.15 mm/s, which corresponded to a strain rate of 0.006 s^−1^ given that the average resting length of clamped membranes was 22 mm. At each strain, a phase and fluorescence picture was taken. When the maximum strain was reached, a similar procedure was followed on the way back down to 0% strain. These trials took about 40 minutes on average to complete. For stretch-relax trials, cells were strained as described above, but relaxed and imaged after each incremental strain was reached. Values of “micrometer strain” do not correspond to the strain in the rubber substrate or the strain exhibited by the cells, due to unavoidable slippage of the membrane from the clamps and partial loss of adhesion of the cells to the substrate. The relationship between micrometer strain and cell and nuclear strain are reported in Figure S1. Note that the slope of the cell strain curve is about double that for nuclear strain, suggesting that there is incomplete force transmission between the basal parts of the cell and the nucleus and/or the nucleus is stiffer than the rest of the cell. In all figures, the strain reported is cell strain as measured using fiducial markers at the cell edges.

### Cell viability

Viability of 80–90% confluent keratinocytes after uniaxial stretch treatments of 0% (control), 69%, and 138% was assessed using the vital inclusion dye fluorescein diacetate (FDA)[33] and the vital exclusion dye 4–6-diamidino-2-phenylindole (DAPI)[34]. After stretching at a rate of 1.3%/sec, cells were stained for one minute using 0.01 mg/mL FDA dissolved in media, followed by three rinses with PBS. Cells were next stained for one second with 3.6 mM DAPI in PBS, followed by three rinses with PBS. The elapsed time the cells were outside the 37°C incubator was 10 minutes which included stretching, staining and imaging. Cells that stained with FDA and excluded DAPI were considered viable, whereas cells that excluded FDA and stained with DAPI were considered necrotic. For the most part, staining with FDA and DAPI were mutually exclusive. Cells that appeared to stain with neither or both dyes (about 1% of cells) were excluded from the cell counts. Viability data were collected from six independent cell stretching trials for each treatment and a total of 32,437 cells were counted and scored.

### Transmission electron microscopy

We prepared keratinocytes for TEM using a modification of the method used by Russell et al. (2004). Adherent cells were subjected to either a control treatment (no stretch), a stretch treatment (average cell strains of 48%, 89%, 126%, and 159%), or a stretch and release treatment (same strains as above, but then relaxed after being held at the target strain for fifteen minutes). The cells were then fixed and embedded while still clamped into the cell stretcher. Cells were fixed for 30 minutes in PBS (pH 7.3) containing 1.5% glutaraldehyde and 1.5% paraformaldehyde and then rinsed three times with 0.1 M sodium cacodylate buffer (pH 7.3). Cells were post-fixed in 1% OsO4 in 0.1 M sodium cacodylate buffer (pH 7.3) for 30 minutes and then washed three times with dH_2_O. Cells were then stained with 1% uranyl acetate, and washed three times with dH_2_O. Cells were dehydrated with ten minute washes of the following EtOH series: 30%, 50%, 70%, 95%, followed by three ten minute washes with absolute EtOH. Cells were infiltrated with 1:1 Polybed:absolute EtOH overnight followed by three thirty minute periods in 100% Polybed. The entire cell stretcher with its clamped membrane covered in Polybed was then placed in a 60°C oven for 48 hours to polymerize the resin. Polymerized strips containing a monolayer of the embedded cells were then peeled off the silastic membrane and mounted onto resin blanks for subsequent trimming and sectioning. Thin sections were cut on an ultramicrotome (Ultracut, Reichert-Jung) and collected on copper grids. Sections were counterstained with saturated uranyl acetate and lead citrate and viewed on a Phillips 300 TEM at 60 kV that was calibrated with a diffraction grating replica with line spacings of 0.463 µm.

### Image Analysis and Statistics

The tortuosity and wavelength of intermediate filament bundles was measured from fluorescence and TEM micrographs using ImageJ v. 1.34s software (NIH). A total of 115 bundles from fluorescence images and a total of 20 bundles from TEM images were used for measuring bundle wavelength in cells that were stretched and relaxed. Tortuosity was calculated as the contour length divided by the end-to-end length and was measured in a total of 200 bundles from fluorescence images and a total of 300 bundles from TEM micrographs. Only bundles that were oriented within 30° on either side of the stretch axis were included in this analysis to eliminate the complication that filaments oriented orthogonal to the stretch axis experienced compression instead of tension. Due to our modest sample sizes for each stretch treatment, we pooled the TEM tortuosity data into two groups–bundles from cells that were fixed in the stretched state (at strains of 48%, 89%, 126%, and 159%) and those that were fixed after being allowed to relax after stretching to the same strains as above. The tortuosity data for these two groups was compared using a two-tailed t-test. Bundle wavelength was measured using both fluorescence and TEM micrographs, but we based our mechanical analysis on the values obtained via TEM, since the fluorescence measurements were significantly larger and likely to be biased towards bundles with larger wavelength buckles due to the limited resolution of light microscopy. Wavelength was measured in 20 bundles from TEM images.

## Supporting Information

Figure S1 Relationship between micrometer strain (measured from clamp to clamp on the cell stretcher), cell strain (circles, measured from cell edge to cell edge) and nuclear strain (squares, measured from opposing edges of the nucleus) in adherent NEB-1 keratinocytes grown and stretched on silastic membranes. Scale bars are SD.
(0.78 MB TIF)
