# Supplementary figures and images for: The Intermediate Filament Network in Cultured Human Keratinocytes Is Remarkably Extensible and Resilient
Source: PLoS One. 2008 Jun 4;3(6):e2327. doi: 10.1371/journal.pone.0002327 (PMC2390850; doi:10.1371/journal.pone.0002327)

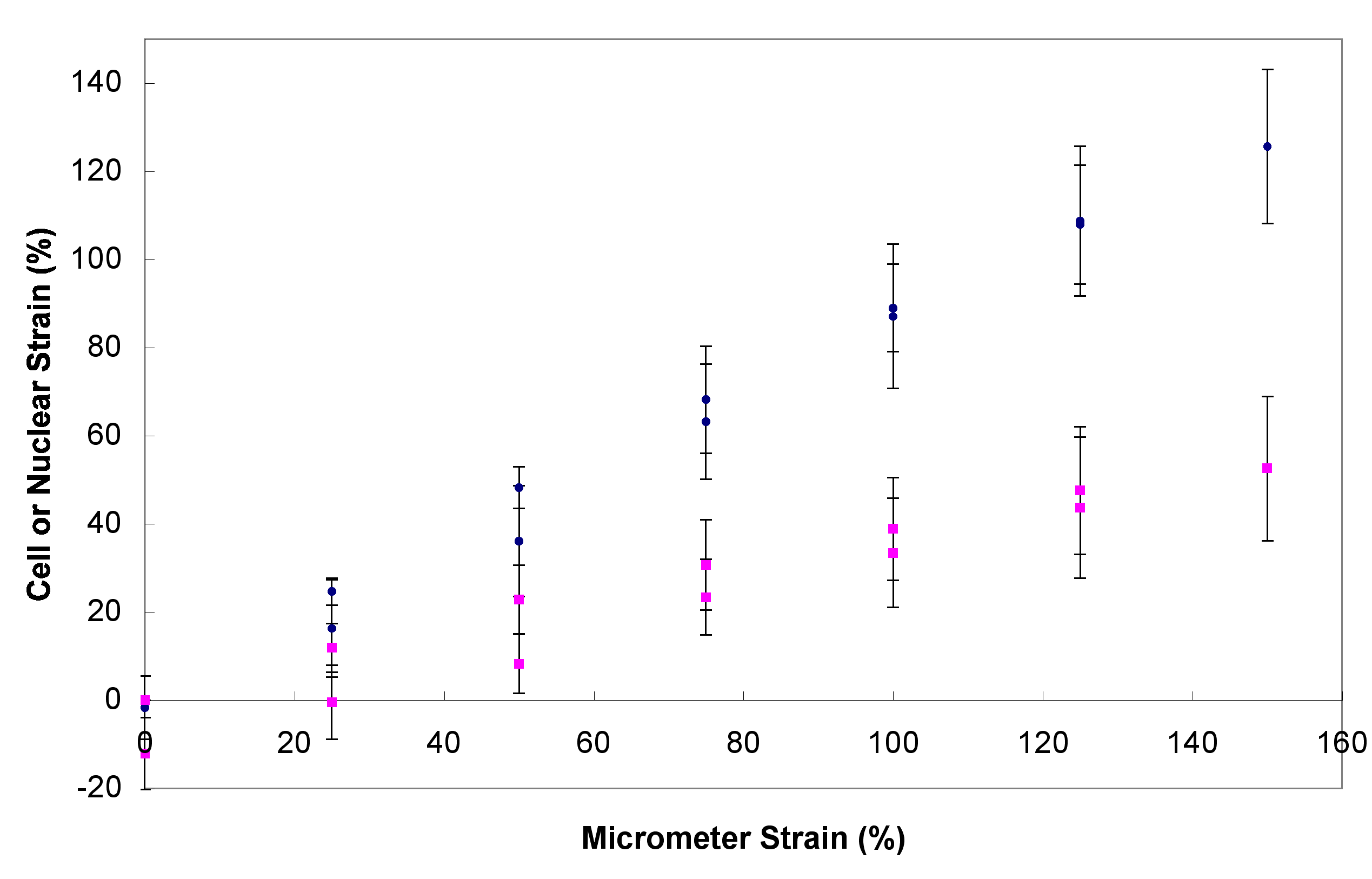

Supplement: Figure S1 — Relationship between micrometer strain (measured from clamp to clamp on the cell stretcher), cell strain (circles, measured from cell edge to cell edge) and nuclear strain (squares, measured from opposing edges of the nucleus) in adherent NEB-1 keratinocytes grown and stretched on silastic membranes. Scale bars are SD. (0.78 MB TIF) [file pone.0002327.s001.tif]
